# Factors Influencing Copulation Duration in *Dastarcus helophoroides* (Fairmaire) (Coleoptera: Bothrideridae)

**DOI:** 10.3390/insects15020104

**Published:** 2024-02-02

**Authors:** Hui-Hui Zhong, Chao-Qun Li, Jiang-Tao Zhang, Li-Feng Wei, Xing-Ping Liu

**Affiliations:** Key Laboratory of State Forestry and Grassland Administration on Forest Ecosystem Protection and Restoration in Poyang Lake Watershed, College of Forestry, Jiangxi Agriculture University, Nanchang 330045, China; zhh@stu.jxau.edu.cn (H.-H.Z.); lcq@stu.jxau.edu.cn (C.-Q.L.); jiang_tao_zhang@163.com (J.-T.Z.); lifengwei102@163.com (L.-F.W.)

**Keywords:** copulation duration, physiological factors, ecological factors, reproductive outputs, *Dastarcus helophoroides* (Fairmaire)

## Abstract

**Simple Summary:**

The copulation duration is one of the important behavioral traits, as it has been proven to be directly correlated with reproductive fitness in insects. Comprehensive knowledge of the factors influencing the copulation duration is often essential for the mass production of natural insect enemies. This study sought to evaluate the role of the copulation duration on reproductive output and to assess the variation in the copulation duration under various physiological and ecological conditions in a laboratory setting, utilizing an ectoparasitic beetle, *Dastarcus helophoroides*, as an experimental model. We found that the copulation duration was highly variable and positively correlated with reproductive fitness. Multiple factors, including body size, mating history, kinship, sex ratio, mating sequence, feeding status, ambient temperature, photoperiod, and time of day, acted on the copulation duration. These results not only augment our understanding of the factors influencing the reproductive fitness of this ectoparasite but may also help to enhance their reproductive potential in a mass-rearing scheme.

**Abstract:**

The gregarious ectoparasitic beetle *Dastarcus helophoroides* (Fairmaire) is considered a primary biocontrol agent for controlling several cerambycid pests in East Asian countries. A thorough study of reproductive behavior is a prerequisite for the mass production of natural insect predators. Nonetheless, little attention has been given to this ectoparasitic beetle. We performed a series of trials to assess whether the adult copulation duration, a key behavioral trait, is differentially influenced by physiological and ecological factors, including body size, mating history, kinship, sex ratio, mating sequence, feeding status, ambient temperature, photoperiod, and time of day. Additionally, the effect of the copulation duration on the reproductive output of this beetle was also investigated. The results indicated that the copulation duration varied considerably, ranging from 1.12 min to 16.40 min and lasting for an average of 9.11 ± 0.12 min. Females with longer copulations laid more eggs and had a greater proportion of eggs hatched. Medium-sized individuals copulated significantly longer than small- and large-sized individuals. The copulation durations were significantly longer when both sexes experienced an asymmetric mating history than when both sexes experienced a symmetric mating history. Inbred couples copulated significantly longer than outbred couples. In terms of the adult sex ratio, increasing the density of females (polygamous group) or males (polyandrous group) led to significantly longer copulation durations than those in the monogamous group. The copulation durations gradually decreased with increasing the mating sequence and temperature. Food-absence couples copulated significantly longer than food-presence couples. The mean copulation duration of the scotophase was significantly longer than that of the photophase. These results demonstrate that all of the analyzed factors emerge as important factors influencing the copulation duration, ultimately affecting the reproductive outputs in this ectoparasitic beetle.

## 1. Introduction

The copulation duration is one of the behavioral traits involved in the mating behavior of insect taxa [1,2]. Currently, it has received attention in studies of behavioral ecology and evolutionary biology, owing to its importance in sexual conflict and sexual selection [3]. Previous studies have shown that the copulation duration is a key component of reproductive fitness in insects, especially in species where the amount of sperm transferred is proportional to the copulation duration; longer copulations often result in greater fecundity and greater fertility, and as a consequence, increase their reproductive potential [4,5,6,7].

A large number of empirical studies have shown that the copulation duration is highly variable between and within insect species [8,9], and how the copulation durations change is a complex and intriguing question. A variety of physiological and ecological factors affect the copulation duration in insects [7,10]. For example, body size plays an important role in driving the copulation duration [11]. Mating history [12,13], age at mating [14,15], and kinship [16,17] have significant effects on the copulation duration. In addition, temperature [18], time of day [19,20], photoperiod [21], mating sequence [13,22], sex ratio or population density [9,23], and feeding status [24,25] also strongly impact the copulation duration in insects. Understanding the factors influencing the variation in the copulation duration will often be essential for adequately understanding sexual selection in insect species [20]. Insects, especially natural insect predators, hold great economic and ecological importance in agroforestry systems, making it critical to analyze the factors impacting their copulation duration and reproductive fitness [26]. However, little attention has been given to the copulation duration by these insect species [25,27].

The gregarious ectoparasitic beetle, *Dastarcus helophoroides* (Fairmaire) (Coleoptera: Bothrideridae), is considered a primary biocontrol agent for controlling several cerambycid pest species [28,29]. This beetle is widely distributed in East Asian countries, including China, Korea, and Japan [28,30,31]. In the field, female *D. helophoroides* usually lay egg clutches near the elliptical holes and on the frass of cerambycid larvae [28]. Newly hatched larvae parasitize the alive larvae and pupae of cerambycid beetles. The developmental duration of this ectoparasitic beetle from egg to pupae is approximately 46.7 days, and the adult life cycle lasts more than four years [32]. Adult *D. helophoroides* are typically nocturnal and positively phototactic insects [33,34]. Currently, a commercialized population of *D. helophoroides* has been widely used in East Asia to effectively suppress the population of the Japanese pine sawyer, *Monochamus alternatus* Hope (Coleoptera: Cerambycidae), a key vector of the lethal pine wilt disease [35,36]. A thorough study of the reproductive behavior of *D. helophoroides*, an effective biocontrol agent, is necessary for its mass production. However, the factors affecting the mating behavior of laboratory-reared strains have received relatively little attention.

We conducted a series of trials to observe the mating behavior of *D. helophoroides*. The purpose of this study was to (1) clarify the length of the copulation duration in constant laboratory conditions; (2) evaluate the effect of the copulation duration on their reproductive output, including fecundity and fertility; and (3) assess the morphological, physiological, and ecological factors that affect the copulation duration. We selected the adult body size, mating history, kinship, sex ratio, mating sequence, feeding status, ambient temperature, photoperiod, and time of day as possible factors influencing the copulation duration. Our results not only augment our understanding of the factors influencing the reproductive fitness of this ectoparasitic beetle but also provide help for mass rearing.

## 2. Materials and Methods

### 2.1. Establishment of Stock Culture

The laboratory colony of *D. helophoroides* used was established in 2014. The initial adults were wild-collected from dead Masson pine trees, *Pinus massoniana* Lamb (Pinales: Pinaceae), located within the area of Ganzhou, Jiangxi Province, China. The collected individuals were subsequently transferred to transparent plastic breeding boxes (length × width × height = 20 × 15 × 10 cm) at a density of sixty individuals per box. The breeding box containing adults was then maintained at 24 ± 1 °C, a relative humidity of 65 ± 5%, and a 14 h light and 10 h dark photoperiod (lights on from 06:00 to 20:00 h and off from 20:00 to 06:00 h) in climate incubators (RDN-400C-4, Southeast Instrument Co., Ltd., Ningbo, China). We provided an artificial diet [37] and distilled water daily. The colony was cultured for 16 successive generations following the standard procedure of egg collection and larvae rearing described by Shi et al. [38]. To avoid inbreeding depression, wild-collected individuals were added to the laboratory colony every year. To prevent mating prior to the trials, newly emerged adults of the 16th generation were introduced and reared individually in transparent plastic Petri dishes (diameter: 7 cm, height: 1.6 cm) lined with dry filter paper. Petri dishes containing experimental insects were placed into the climate incubators and kept under the same ambient conditions. Thirty-day-old virgin adults were used in all trials, as *D. helophoroides* become sexually mature approximately one month after emergence under similar ambient conditions.

To harvest individuals with the same close kin, twenty couples were randomly selected from the colony of the 15th generation and transferred separately into plastic Petri dishes (size as above), and egg collection and larval rearing were conducted as described above. The progeny produced by each couple represented one full-sib family. Newly emerged beetles from twenty families were separated and raised individually in a plastic Petri dish. Thirty-day-old sexually mature virgin adults from each family were used in the kinship trial.

### 2.2. Mating Condition

Prior to the beginning of each trial, all 30-day-old sexually mature virgin adults were randomly selected and sexed under a stereomicroscope (SZ60, Olympus, Tokyo, Japan), as described by Tang et al. [39]. These sexed adults were then paired individually in separate plastic Petri dishes containing food and water except for the feeding status trial. For all the experiments, the males were first introduced into each Petri dish and allowed to feed for 10 min before the females were added. The Petri dishes containing couples were transferred into the behavioral observation chamber under the same ambient conditions as those described above. The mating behavior of each couple was monitored using a digital video camera (high definition, 720P, 1/2.7″ Sony CCD camera, Tokyo, Japan). After monitoring was complete, we checked every video and recorded the copulation duration. The copulation duration was measured from the insertion of the male’s aedeagus into the female’s genitalia to the extraction of the male’s aedeagus. Unless otherwise specified, all mating experiments were monitored for one week. All trials were performed in Petri dishes.

### 2.3. Trial 1: Copulation Duration Observation

To determine the average copulation duration of *D. helophoroides* individuals, one thousand 30-day-old sexually mature adults were randomly selected and paired individually. The couples were allowed to mate freely. The copulation duration of the first mating of each couple was registered in seconds. If no copulation occurred within one week after the observation began, the couples were discarded, and the data were excluded from the analysis.

### 2.4. Trial 2: Effect of Copulation Duration on Reproductive Outputs

To estimate the possible role of the copulation duration in *D. helophoroides*, we used the total number of eggs (fecundity) and the proportion of eggs hatched (fertility) as indicators of the female reproductive traits for different copulation durations. Only females that had mated once in trial 1 were selected and reared individually in Petri dishes. We provided an artificial diet and distilled water daily as a food source and kraft paper as an oviposition substrate. The egg clutches of each mated female were transferred, along with the kraft papers, to an empty Petri dish. The number of eggs was recorded every day under a stereomicroscope (SZ60, Olympus, Tokyo, Japan) for eight weeks. Eggs were observed for hatching one week after laying. Eggs that did not hatch during this period were considered infertile. In this trial, 137 mated females with different copulation durations were selected as observational samples.

### 2.5. Trial 3: Factors That Affect the Copulation Duration

#### 2.5.1. Effect of Body Size

Prior to the beginning of the trial, 30-day-old sexually mature virgin adults were subjected to measurements of body length using an electronic digital Vernier caliper with an accuracy of 0.01 mm (DL91300, Deli Tools Co., Ltd., Ningbo, China). These insects were then arranged into large, medium, and small size groups according to their body length. The difference between the three groups was statistically significant (ANOVA, Females: *F* = 896.142, *df* = 2, 225, *p* < 0.001; Males: *F* = 752.526, *df* = 2, 225, *p* < 0.001). Nine possible pairing treatments involving coupling adults from each of the three body-size groups were established. The copulation duration of the first mating was recorded in seconds. In this trial, each Petri dish served as a replicate, and each pairing treatment was replicated 25 times.

#### 2.5.2. Effect of Mating History

We divided the virgin adults into two groups. In the first group, virgin males and virgin females were paired individually, and their mating behavior was monitored for 24 h. Once copulation ended, the copulated males and females were isolated in new Petri dishes. These adults in the 24 h prior to an experiment were designated as previously mated individuals. In the second group, the adults held in their original Petri dishes without mating were designated as virgin individuals. On the day of the experiment, pairs of virgin and mated adults were subjected to mating via the following four treatments: virgin female × virgin male, virgin female × previously mated male, previously mated female × virgin male, and previously mated female × previously mated male. Each treatment was replicated 35 times. All replicates were allowed to copulate only once, and their copulation duration was analyzed.

#### 2.5.3. Effect of Kinship

To measure the effect of kinship on the copulation duration of *D. helophoroides*, 30-day-old sexually mature virgin adults from each of the twenty families were randomly selected as experimental insects. The experiment consisted of the following two treatments: (1) an inbred group, i.e., a virgin female or male paired with a full-sibling virgin partner, and (2) an outbred group, i.e., a virgin female or male from one family paired with a non-sibling virgin partner from another family. Males and females from each of the twenty families were randomly assigned to one of two mating treatments. A total of 100 male–female pairs were established, and 88 single copulations occurred. The copulation durations were registered, and we compared the differences between the inbred and outbred groups.

#### 2.5.4. Effect of Sex Ratio

To determine whether the variation in copulation duration is related to the changes in the sex ratio, we established three sex ratio treatments. In the monogamous group, one virgin female was paired with one virgin male (one female/one male). In the polyandrous group, one virgin female was paired with three virgin males (one female/three males). In the polygamous group, three virgin females were paired with one virgin male (three females/one male). We timed the copulation duration as soon as a mating began in each treatment. Each Petri dish served as a replicate, and each treatment was replicated 40 times.

#### 2.5.5. Effect of Mating Sequence

To test the relationship between the copulation duration and the mating sequence, a total of sixty sexually mature virgin males and females of the same age were paired individually. Mating pairs in each Petri dish were monitored for one month. The copulation duration of each mating in each pair was registered in seconds.

#### 2.5.6. Effect of Feeding Status

We established four treatments to determine the effect of feeding status on the copulation duration. In the first group, couples were provided with an artificial diet and distilled water ad libitum daily. In the second group, couples were provided only an artificial diet. In the third group, couples were provided with only distilled water. In the fourth group, couples were provided with neither food nor water. Each of the four treatments was replicated 40 times. The mating behavior of each pair was observed for one week, and the copulation duration of the first mating was recorded.

#### 2.5.7. Effect of Ambient Temperature

To test the effect of temperature on the copulation duration, the Petri dishes with paired adults were transferred to a temperature-controlled behavioral observation chamber held at 21 °C, 24 °C, 27 °C, and 30 °C for one week, respectively. This temperature range was chosen because previous experiments have shown that the behavioral activity and fecundity of this species are drastically reduced when the ambient temperature is less than 21 °C or greater than 28 °C [40]. The various temperatures were used as treatments, and each treatment was replicated 40 times. The copulation duration of the first mating in each replicate was recorded and compared across the temperature range.

#### 2.5.8. Effects of Photoperiod and Time of Day

To determine the variation in the copulation duration between the photophase and scotophase and the time of day at mating, the data on the copulation duration in trial 1 were used for further analysis. We categorized the data of the copulation duration according to the time of day and photoperiod of mating occurrence, respectively. We then compared the difference in copulation duration between the photophase and scotophase and the time of day.

### 2.6. Data Analysis

All the statistical analyses were conducted using SPSS 19.0 software (SPSS, Inc., Chicago, IL, USA). The data were checked for normality and heterogeneity of variance following Kolmogorov–Smirnov’s and Bartlett’s tests, respectively. Descriptive statistical procedures were performed to describe the copulation duration. Regression analysis was subsequently performed to test the relationship between copulation duration and fecundity or fertility. The proportional data were subjected to arcsine-square-root transformation before analysis. The data on the sex ratio, mating sequence, ambient temperature, and time of day were analyzed using the nonparametric Kruskal–Wallis test or one-way ANOVA followed by Tukey’s HSD test if these data failed or passed the normality of distribution and homogeneity of variances, respectively. The effects of body size, mating history, and feeding status on the copulation duration were evaluated using two-way ANOVA with treatment and sex as the factors in body size and mating history trials, and with diet and water as the factors in the feeding status trials. The data on kinship, mating history, and photoperiod were analyzed using an independent samples *t*-test. The results are presented as the means ± standard errors (SEs).

## 3. Results

### 3.1. Copulation Duration and Its Effect on Reproductive Outputs

**Figure 1:**
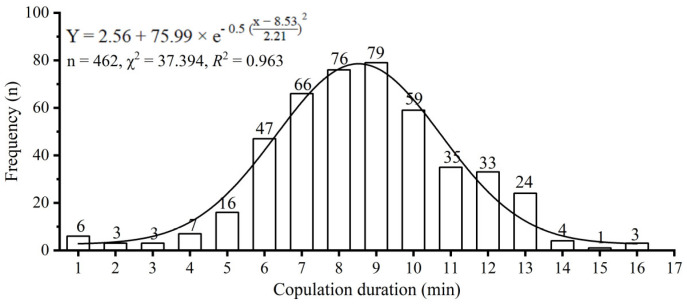
Frequency of the copulation duration in *Dastarcus helophoroides*. The number on each bar is the frequency of copulation duration.

**Figure 2:**
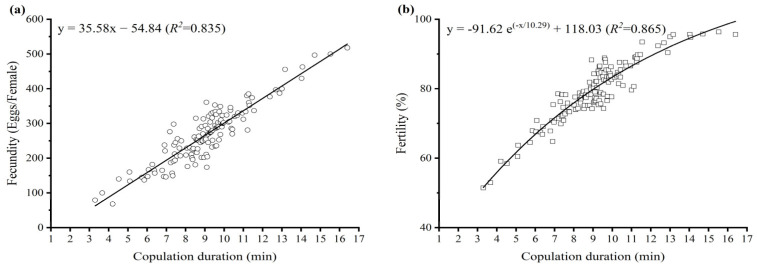
Relationship between copulation duration and reproductive output: (**a**) fecundity and (**b**) fertility in *Dastarcus helophoroides* during an 8-week observation period.

The copulation duration was recorded for 462 couples of *D. helophoroides*. The shortest copulation duration was 1.12 min, and the longest duration was 16.40 min. The mean copulation duration was 9.11 ± 0.12 min (Figure 1). Females with longer copulations laid more eggs and had a greater proportion of eggs hatched than females with shorter copulations. Regression analysis revealed that there was a highly significant positive relationship between copulation duration and egg production (Pearson’s correlation, *r* = 0.914, *p* < 0.001, Figure 2a) and between copulation duration and egg hatchability (Spearman’s correlation, *p* = 0.873, *p* < 0.001, Figure 2b).

### 3.2. Influence Factors Influencing the Copulation Duration

#### 3.2.1. Body Size

**Figure 3:**
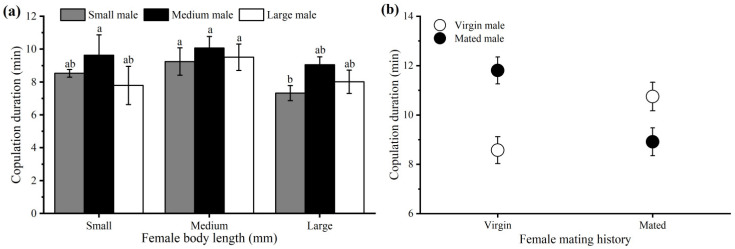
Effect of body size (**a**) and mating history (**b**) on copulation duration in *Dastarcus helophoroides*. Bars with the same lowercase letter are not significantly different at the *p* < 0.05 level (Tukey’s test).

Copulation durations were significantly related to body size. Medium-sized individuals copulated longer (9.69 ± 0.33 min) than did small-sized (8.47 ± 0.40 min) and large-sized individuals (8.23 ± 0.34 min). Almost an identical copulation duration was observed for the male and female mating combinations (Figure 3a). Two-way ANOVA revealed a highly significant effect of body size (*F* = 5.320, *df* = 2, 148, *p* = 0.006), whereas there was no effect of sex (*F* = 0.045, *df* = 2, 148, *p* = 0.833) or body size × sex interaction (*F* = 0.206, *df* = 2, 148, *p* = 0.814) on the copulation duration.

#### 3.2.2. Mating History

Previously mated individuals copulated longer than virgin individuals did (10.11 ± 0.31 min and 9.90 ± 0.30 min, respectively). The mean copulation durations for females and males were 10.00 ± 0.30 min and 10.01 ± 0.30 min, respectively. There was no significant effect of sex (*F* = 0.000, *df* = 1, 226, *p* = 0.991), mating history (*F* = 0.250, *df* = 1, 226, *p* = 0.618), or sex × mating history interaction (*F* = 1.900, *df* = 1, 226, *p* = 0.169) on the copulation duration (Figure 3b). Independent sample *t*-tests revealed that the copulation durations were significantly greater when both sexes experienced an asymmetric mating history than when both sexes experienced a symmetric mating history (*p* < 0.01).

#### 3.2.3. Sex Ratio

**Figure 4:**
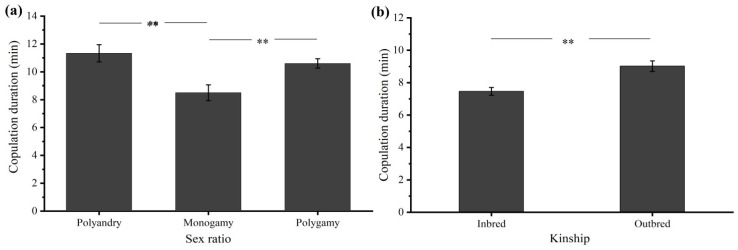
Effect of sex ratio (**a**) and kinship (**b**) on copulation duration in *Dastarcus helophoroides*. A double asterisk denotes a significant difference (*p* < 0.01, independent samples *t*-test).

The sex ratio had a significant effect on the copulation duration (*F* = 7.896, *df* = 2, 81, *p* = 0.001). In the polyandrous group, copulation with virgin females lasted 11.33 ± 0.61 min, but only 8.50 ± 0.57 min in the monogamous group (*p* = 0.001, Figure 4a). The mean copulation duration in the polygamous group (10.60 ± 0.34 min) was also significantly longer than that in the monogamous group (*p* = 0.012, Figure 4a).

#### 3.2.4. Kinship

The kinship of couples had a significant effect on the copulation duration. The mean copulation durations for the inbred and outbred couples were 7.46 ± 0.24 min and 9.02 ± 0.33 min, respectively. The independent sample *t*-test revealed that kinship had a significant effect on the copulation duration (*t* = −3.836, *df* = 86, *p* < 0.001, Figure 4b).

#### 3.2.5. Mating Sequence

**Figure 5:**
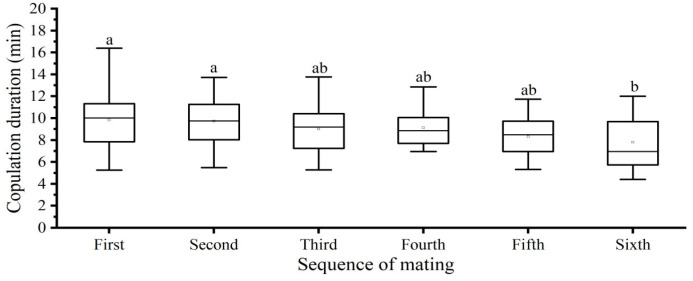
Effect of mating sequence on copulation duration in *Dastarcus helophoroides*. The line and square frame inside the boxes indicate the medians and means, respectively; the heights of the boxes indicate the first and third quartiles, and the whiskers indicate the data range. Different letters are significantly different at the *p* < 0.05 level (Tukey’s test).

In this trial, 47 couples were found to have completed at least one and, at most, six matings during the one-month observation period. The copulation duration varied significantly and gradually decreased with increasing the mating sequence. The first copulation had a mean duration of 9.83 ± 0.35 min, whereas the fifth copulation lasted for 8.32 ± 0.42 min and the sixth for 7.81 ± 0.73 min. Post hoc comparisons of these data showed that the first copulation was significantly longer than the sixth copulation (*F* = 2.908, *df* = 5, 168, *p* = 0.015, Figure 5).

#### 3.2.6. Feeding Status

The copulation duration was slightly longer in the water-absence treatment (10.15 ± 0.32 min) than in the water-presence treatment (9.42 ± 0.31 min), but no statistically significant difference was detected (*F* = 2.758, *df* = 1, 99, *p* = 0.100). However, the copulation duration was significantly longer in the food-absence treatment (10.37 ± 0.31 min) than in the food-presence treatment (9.20 ± 0.32 min) (*F* = 6.897, *df* = 1, 99, *p* = 0.010). There was also a significant effect of the food × water interaction (*F* = 10.003, *df* = 1, 99, *p* = 0.002) on the copulation duration.

#### 3.2.7. Ambient Temperature

**Figure 6:**
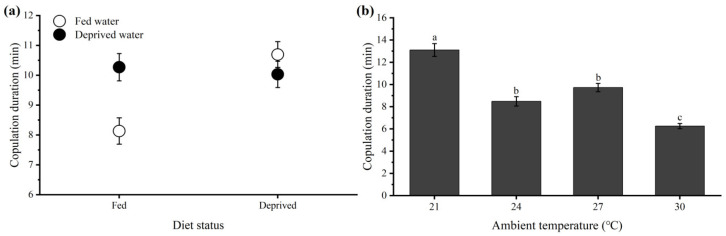
Effect of adult feeding status (**a**) and ambient temperature (**b**) on copulation duration in *Dastarcus helophoroides*. Different letters above bars are significantly different at the *p* < 0.05 level (Tukey’s test).

Figure 6b shows the copulation duration at four different constant temperatures. As the temperature increased, the duration of copulation decreased from 13.11 ± 0.58 min at 21 °C to 6.26 ± 0.23 min at 30 °C. Statistical analyses clearly revealed that the copulation duration was significantly negatively correlated with the ambient temperature in *D. helophoroides* (Kruskal–Wallis test, *H* = 63.010, *df* = 3, *p* < 0.001).

#### 3.2.8. Photoperiod and Time of Day

**Figure 7:**
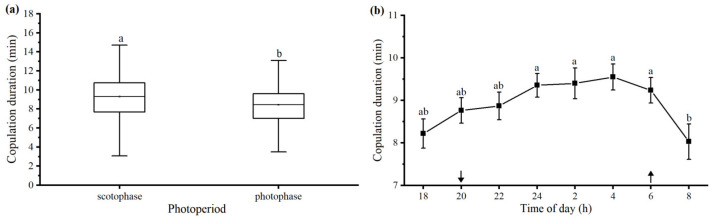
Effect of photoperiod (**a**) and time of day (**b**) on copulation duration in *Dastarcus helophoroides*. Downward and upward arrows indicate the times of lights off (20:00 p.m.) and lights on (6:00 a.m.); different letters are significantly different at the *p* < 0.05 level (independent samples *t*-test and Tukey’s test, respectively).

Most of the observed copulations occurred during the scotophase, and no matings occurred from 8:00 AM to 16:00 PM. The mean copulation duration during the scotophase (9.29 ± 0.14 min) was significantly longer than that during the photophase (8.44 ± 0.20 min) (*t* = 3.493, *df* = 459, *p* = 0.001, Figure 7a). The copulation duration varied considerably with the time of day, increasing from 8.22 ± 0.34 min at 16:00–18:00 p.m. to 9.55 ± 0.31 min at 2:00–4:00 a.m. and then declining to 8.03 ± 0.42 min at 6:00–8:00 AM. There was a statistically significant difference in the copulation duration among the time of day (*F* = 2.047, *df* = 7, 453, *p* = 0.048, Figure 7b).

## 4. Discussion

Our experiments generated three clear findings. First, the copulation duration in *D. helophoroides* is highly variable under constant laboratory conditions. Second, there is a significant effect of the copulation duration on reproductive fitness, as fecundity and fertility are positively correlated with the copulation duration in *D. helophoroides*. Finally, the copulation duration in *D. helophoroides* is influenced by multiple physiological and experimental factors, including body size, mating history, kinship, sex ratio, mating sequence, feeding status, ambient temperature, photoperiod, and the time of day.

As an important behavioral trait, copulation duration has received much attention in the field of reproductive behavioral ecology. Earlier studies have revealed that the copulation duration in insects varies considerably between and within species, ranging from several seconds to a few minutes [8] and several hours [4,9], even up to 79 days [41]. In contrast, in our observation, the copulation duration of *D. helophoroides* ranged from 1.12 min to 16.40 min and lasted an average of 9.11 ± 0.12 min (Figure 1). The most recent reports suggest that shorter copulation durations can achieve benefits by reducing the risk of predation and directly engaging in oviposition [42].

The length of the copulation duration has a profound effect on reproductive fitness in insects [43]. Two hypotheses have been proposed to explain the benefits of long-lasting copulations [3]. The ejaculate transfer hypothesis stipulates that females with longer copulations may produce significantly more offspring in species where the copulation duration is proportional to the transfer of sperm [7]. This positive correlation between copulation duration and reproductive fitness indicates that the seminal substances that increase female egg production are regulated by the copulation duration [5]. However, in other insect species, the copulation duration does not correlate with female fecundity or fertility [19]. The extended mate-guarding hypothesis may be the best explanation for this difference [27]. In the present study, all mated females laid fertilized eggs regardless of the duration of copulation. However, a short copulation duration was not sufficient for fertilizing all the eggs produced, possibly because the females did not receive sufficient sperm. In addition, fecundity and fertility in *D. helophoroides* significantly increased with a protracted copulation duration (Figure 2). The increase in reproductive output may be directly related to more sperm being transferred, which supports the ejaculate transfer hypothesis. These results are consistent with earlier studies of other coleopteran, dipteran, and heteropteran species [6,21,44]. According to our observation, male and female *D. helophoroides* were paired in opposite straight-line positions during copulation and separated once they had finished mating. Therefore, we did not find any postcopulatory guarding behavior. Moreover, we cannot confirm whether females who laid more fertilized eggs received more sperm because we did not dissect the females who experienced different copulation durations. Additional work is needed to reveal the role of sperm in female reproductive fitness.

Variation in copulation duration is influenced by several physiological and ecological factors [7,45,46]. Many insect species exhibit a positive correlation [12], a differential effect between body size and copulation duration [10,47], or a seasonal dependence, i.e., the copulation duration is positively correlated with body size in the earlier part of the year but negatively correlated with body size in the late part of the year [11]. The results from our present trial suggested that medium-sized individuals copulated significantly longer than did smaller and larger individuals, but no significant effect of sex was observed on the copulation duration of *D. helophoroides* (Figure 3a). It is probable that medium-sized adults are more fecund than adults of other body sizes, resulting in sexual selection for medium-sized adults. This result is consistent with that of a previous study on *Drosophila melanogaster* [47]. Alternatively, the longer copulation duration in medium-sized adults might be attributed to the fact that medium-sized individuals are “genitalia compatible” in all combinations, i.e., with both small and large individuals [48]. A similar result has been observed in the stag beetle *Lucanus cervus*, where the largest males were unable to mate with smaller females [49]. However, insect body size is influenced by genetics, nutrition, ambient temperature, or a combination of these factors. It is difficult to define how much of the variation is genetic and how much is environmental in any given species [47].

Mating history was also reported to affect the copulation durations in insect species. For some species, virgin individuals copulate for a longer duration than mated individuals [2], whereas for others, the opposite result is shown [4,13,45]. In contrast, we found that the duration of copulation was not significantly affected by the mating history of either sex in *D. helophoroides*. However, the copulation duration was significantly longer when both sexes experienced an asymmetric mating history than when both sexes experienced a symmetric mating history (Figure 3b). The reason for this discrepancy remains unclear. Similar results were also reported in other insect species [12,45,50]. Previous studies have suggested that the kinship of one’s partner has a significant effect on the copulation duration [16,17]. We found that *D. helophoroides* females spent significantly more time copulating with unfamiliar males than with their full-sibling brothers (Figure 4b). This result suggested that adult *D. helophoroides* can recognize the relatedness of their partners and initiate inbreeding avoidance by decreasing the copulation duration. A similar result was also reported for the maize weevil *Sitophilus zeamais* [51].

In the context of a male-biased sex ratio, male–male competition is expected to increase, and females become choosier [23,52]. Under these circumstances, males may prolong their copulations to reduce the chance of sperm competition and sperm displacement [53]. This phenomenon has been widely previously observed in many insect species, such as the damselfly *Ceriagrion tenellum* [4] and the hematophagous bug *Triatoma brasiliensis* [54]. According to our observation, the copulation duration of *D. helophoroides* was longer in the presence of multiple males (polyandrous group) compared with the observations of isolated pairs (monogamous group) (Figure 4a), suggesting that male disturbance is used as a cue to prolong copulation. Moreover, with a female-biased sex ratio, females may become less choosy and less likely to reject male mating attempts, leading to a longer male copulation duration. A similar result was reported for the seed bug *Nysius huttoni* [53].

The duration of copulation varies significantly according to the mating sequence of insect species with multiple mating. For *D. helophoroides*, we recorded up to six matings during a one-month observation period. Our results clearly showed that this ectoparasitic beetle could mate multiple times and that males could produce new sperm over time, although the lifetime mating frequency of this insect is not known. Moreover, the copulation durations gradually decreased with increasing the mating sequence (Figure 5). The same pattern was observed for the African squinting bush brown butterfly *Bicyclus anynana* [22] and the hematophagous bug *Triatoma brasiliensis* [54]. A possible explanation for this result is that the amount of sperm transferred gradually decreases during consecutive mating.

Ecological conditions such as access to an adequate and appropriate diet are among the factors that affect insect mating behavior and fitness [24]. Many insects, such as the red flour beetle *Tribolium castaneum* [55], the red palm weevil *Rhynchophorus ferrugineus* [10], and the aphidophagous ladybird beetle *Propylea dissecta* [24], attempt to avoid copulation and even decrease the copulation duration under starvation conditions. However, in some published insects, starvation does not have any meaningful effect on the copulation duration, and the results are not significant, which may indicate that nutritional status is not an important factor affecting mating, such as in the medfly *Ceratitis capitata* [56] and the aphidophagous ladybird beetle *Hippodamia variegata* [25]. Our experiments clearly demonstrated the effects of feeding status on copulation durations, as short-term food-absence individuals copulated for much longer durations than did food-presence individuals (Figure 6a). The female foraging hypothesis possibly provides the best explanation for our finding, which suggests that female *D. helophoroides* may ingest more nutritional ejaculate through extended copulation when female energetic needs are greater [57].

Temperature is an important ecological factor affecting the copulation duration of insect species. Almost all studies have shown that the copulation duration decreases as the temperatures increase. Our current work also revealed that the copulation duration of *D. helophoroides* was strictly determined by the ambient temperature at the time of mating, and lower temperatures during mating resulted in longer copulation durations (Figure 6b). A negative relationship between ambient temperature and copulation duration has been reported for other arthropods, such as the adzuki bean beetle *Callosobruchus chinensis* [46] and the phytoseiid mite *Neoseiulus californicus* [18]. This result may be related to the male’s initiation of mating termination after the completion (or incomplete) of temperature-dependent sperm transfer activity, but additional studies are needed to verify this hypothesis [18]. Moreover, to ensure normal activity and reproduction, the optimal temperature for mass rearing of this parasitoid was above 21 °C and below 27 °C. This temperature range is also consistent with that in a previous study of this beetle [40].

Photoperiod and the time of day in which copulation started have also been confirmed as key factors influencing the copulation duration in various insect species. For example, the copulation duration was negatively correlated with the time of day when copulation started in the damselfly *Ceriagrion tenellum* [4] and the red milkweed beetle *Tetraopes tetrophthalmus* [20]. The mean copulation duration of the photophase was significantly longer than that of the scotophase in the New Zealand bug, *Nysius huttoni* [21]. On the basis of our observations, we also found a clear effect of the time of day and photoperiod on the copulation duration in *D. helophoroides*. However, our findings were not consistent with those for the above-mentioned insects. The copulation duration varied throughout the day, and no mating occurred from 8:00 a.m. to 16:00 p.m. The mean copulation duration in the scotophase was significantly longer than that in the photophase, and the longest copulations occurred at dawn (Figure 7). A reasonable explanation for the longer copulation duration is that the copulation duration is affected by the ambient temperature. Longer copulation durations at dawn or in the scotophase may be the result of slower behavioral rates associated with cooler temperatures [20]. This explanation clearly does not correspond to our present results because we conducted this experiment in a chamber at a constant temperature. However, the photoperiod and the diurnal rhythm during which copulation occurs likely act on this species, but further verification is required [21].

## 5. Conclusions

In conclusion, the present study provides insight into multiple factors affecting the copulation duration of the ectoparasitic beetle *D. helophoroides* and ultimately further affects their reproductive fitness. This knowledge on copulation duration can help us develop suitable strategies for mass rearing of this insect natural predator. For adult *D. helophoroides*, medium-sized and outbred individuals should be collected for group rearing at a lower temperature with sufficient food. These operations may significantly prolong the copulation duration, thereby allowing much more high-quality offspring to be harvested. In addition, the effects of adult age at mating on the copulation duration were also well documented [10,15]. Conducting a relevant study on the effect of adult age at mating on the copulation duration by this ectoparasitic beetle may be very interesting because the lifespan of this insect lasts more than four years [32]. Indeed, numerous studies have shown that prolonged copulation duration does not always increase the reproductive fitness of females. For example, prolonged copulation did not increase the number of fertilized eggs but rather was a function of the ‘postcopulatory’ strategy to prevent subsequent mating of a female [58]. Furthermore, with increasing temperatures, the copulation duration gradually decreases, but the reproductive output significantly increases [59,60]. Thus, additional studies are needed to determine the relationship between the variability in copulation duration due to multiple factors and fitness consequences in *D. helophoroides*.

## Data Availability

All data generated and/or analyzed during the current study are available from the corresponding author upon request.
